# Exsection of Sup. Maxillary Nerve

**Published:** 1879-11

**Authors:** Herbert W. Little

**Affiliations:** House Surgeon; New York


Medical and Surgical Journal.

Vol. XVII.] NOVEMBER—1879. [No. 8.

### Original Communications.

### PRESBYTERIAN HOSPITAL OF THE CITY OF NEW YORK—EXSECTION OF SUP. MAXILLARY NERVE.

By HERBERT W. LITTLE, M.D., House Surgeon.

J. V., æt. 41. Barkeeper. Admitted into the hospital September 25th. Under the care of Dr. William T. White. Patient gives the following history: Mother has been troubled as he is for 59 years, with occasional remission. None of the rest of the family have ever been so affected. Patient has had evidence of phthisis for ten years, but no general trouble.

Six years ago the first molar tooth of left upper jaw ulcerated, causing great pain; tooth was extracted without any relief. Subsequently the two adjoining bicuspids were also removed, without relief. During the past six years he has suffered terribly from facial neuralgia along the distribution of the sup. maxillary nerve, giving him most intense agony for a few minutes at a time. For months at a time he would have complete remissions of the attack, when the old and terrible trouble would return. Pain generally most severe during cold weather.

In January, 1875, the nerve was divided in the German hospital of this city, at the point where it emerges from the infraorbital foramen. For fourteen days only after the operation was he free from pain; no subsequent diminution. Three years ago took arsenic for three months. Has taken bromides and chloral continuously with subcutaneous injections of morphia over the seat of the pain.

Present attack has lasted for six weeks almost without interruption, and has been unusually severe. Can obtain no relief except from morphia.

The patient is a man of very good physique, but suffers terribly from his affliction. Appetite poor. Incapacitated for work. Has lost fifty pounds in weight during the past four years. When admitted, had a severe paroxysm of pain, and has had them every few minutes. The left side of the face over the molar bone presents an indurated spot where he has received hypodermics.

It was decided that his only hope for relief consisted in having an operation performed.

Consequently the day after admittance the patient was etherized, and Dr. Wm. T. White, aided by Dr. A. C. Post, proceeded to operate. An incision was made on the left side of the face, half inch below the inner canthus of the eye, and carried down opposite the ala of the nose. From that point another incision was carried obliquely upward and terminating half inch below the external canthus. This triangular flap was dissected up from the bone, turned up over the eye, held there by a silk ligature. The periosteum was separated from the sup. maxilla below the infraorbital foramen. The infraorbital nerve was dissected out and a piece of ligature tied around the distal extremity. Then the antrum was opened by a trephine; two discs being removed, each half an inch in diameter. This opening was enlarged and made nearly circular by a rouget. The infraorbital foramen was opened with bone forceps. The mucous membrane lining the antrum on each side of the infraorbital canal was divided with the knife. The canal enlarged, the borders being cut away with a small chisel so as to allow the nerve to be drawn down; the posterior wall of the antrum was pierced with a small trephine; the nerve traced back to the foramen rotundum; the nerve divided with curved scissors, the piece removed being two inches in length; there was considerable hemorrhage; six ligatures were applied to vessels in the flap; the bleeding in the bottom of the wound was controlled by pieces of sponge covered with tannin; two pin sutures were put in on each side, bringing together the wound for about half its distance, the rest being left open and lint, wet with carbolic acid, 1 to 40, applied.

The patient recovered well from the effects of the ether and is quite easy. There is no hemorrhage from the wound. During the night was rather restless and had some pain, but not the paroxysms he had before the operation. It was necessary to administer U. S. P. sol. of morphia at different intervals during the day to relieve the pain. Takes nourishment through a glass tube.

On the 29th had a severe paroxysm at 5 P.M. as before the operation. It appeared to be excited by the movement of his jaws while masticating. Three days after last date and six days from the operation, patient is doing extremely well; has but very little pain, eats and sleeps tolerably well, and is in a fair way for complete relief.

